# Deep learning-based predictions of older adults' adherence to cognitive training to support training efficacy

**DOI:** 10.3389/fpsyg.2022.980778

**Published:** 2022-11-17

**Authors:** Ankita Singh, Shayok Chakraborty, Zhe He, Shubo Tian, Shenghao Zhang, Mia Liza A. Lustria, Neil Charness, Nelson A. Roque, Erin R. Harrell, Walter R. Boot

**Affiliations:** ^1^Department of Computer Science, Florida State University, Tallahassee, FL, United States; ^2^School of Information, Florida State University, Tallahassee, FL, United States; ^3^College of Medicine, Florida State University, Tallahassee, FL, United States; ^4^Department of Statistics, Florida State University, Tallahassee, FL, United States; ^5^Department of Psychology, Florida State University, Tallahassee, FL, United States; ^6^Department of Psychology, University of Central Florida, Orlando, FL, United States; ^7^Department of Psychology, The University of Alabama, Tuscaloosa, AL, United States

**Keywords:** artificial intelligence, deep learning, adherence prediction, cognitive training, early detection of cognitive decline

## Abstract

As the population ages, the number of older adults experiencing mild cognitive impairment (MCI), Alzheimer's disease, and other forms of dementia will increase dramatically over the next few decades. Unfortunately, cognitive changes associated with these conditions threaten independence and quality of life. To address this, researchers have developed promising cognitive training interventions to help prevent or reverse cognitive decline and cognitive impairment. However, the promise of these interventions will not be realized unless older adults regularly engage with them over the long term, and like many health behaviors, adherence to cognitive training interventions can often be poor. To maximize training benefits, it would be useful to be able to predict when adherence lapses for each individual, so that support systems can be personalized to bolster adherence and intervention engagement at optimal time points. The current research uses data from a technology-based cognitive intervention study to recognize patterns in participants' adherence levels and predict their future adherence to the training program. We leveraged the feature learning capabilities of deep neural networks to predict patterns of adherence for a given participant, based on their past behavior. A separate, personalized model was trained for each participant to capture individualistic features of adherence. We posed the adherence prediction as a binary classification problem and exploited multivariate time series analysis using an adaptive window size for model training. Further, data augmentation techniques were used to overcome the challenge of limited training data and enhance the size of the dataset. To the best of our knowledge, this is the first research effort to use advanced machine learning techniques to predict older adults' daily adherence to cognitive training programs. Experimental evaluations corroborated the promise and potential of deep learning models for adherence prediction, which furnished highest mean F-scores of 75.5, 75.5, and 74.6% for the Convolution Neural Network (CNN), Long Short-Term Memory (LSTM) network, and CNN-LSTM models respectively.

## 1. Introduction

The US adult population over the age of 65 is growing rapidly and is projected to nearly double in the next 40 years (Mather et al., 2015). As people age, they experience cognitive decline, which can affect their functional independence and the ability to process information to make daily decisions. Such cognitive decline can range from that anticipated in normal aging to the more serious decline of dementia or other related diseases (e.g., Alzheimer's). Finding ways to address this problem can significantly improve the lives of older adults, their families, and reduce care burden and cost of care. A growing body of pharmacological and non-pharmacological intervention studies (Rebok et al., 2014; Rafii and Aisen, 2015; Kramer and Colcombe, 2018) have shown the potential to counteract the effects of age-associated cognitive decline. Cognitive training is one of the most common forms of non-pharmacological interventions. This approach commonly uses guided technology-based exercises, focused on improving specific cognitive functions such as memory, attention, or problem-solving. Although these brain-training interventions are widely used, research on the effectiveness of such interventions has resulted in both positive and neutral outcomes (Bahar-Fuchs et al., 2013; Simons et al., 2016; Chiu et al., 2017; Kallio et al., 2017; Ge et al., 2018; Nguyen et al., 2019; Sala et al., 2019; Turunen et al., 2019; Zhang et al., 2019; Basak et al., 2020; Harrell et al., 2021; Kuo et al., 2021). Adherence is the key to maximize the potential benefits of cognitive training interventions. The WHO defines adherence as “*the extent to which a person's behavior agrees with the recommendations of a healthcare provider”* (Sabaté and Organization, 2001). While various studies suggest that adherence is influenced by cognition, functional ability, and the intervention approach (Sabaté, 2003; Munro et al., 2007; Picorelli et al., 2014; Room et al., 2017; Rivera-Torres et al., 2019; Turunen et al., 2019), it is not yet fully understood why some participants are more adherent to cognitive training programs than others. To improve the potential effects of cognitive training, it may be beneficial to study participants' adherence patterns and identify those who are least likely to adhere to the training process.

Our ongoing Adherence Promotion with Person-Centered Technology (APPT) project aims at understanding long-term adherence barriers, developing algorithms to predict and prevent adherence failures, and ultimately facilitating early detection of age-related cognitive decline and its treatment. Accurate adherence prediction can help in the development of a just-in-time AI based reminder system that will send tailored messages at optimal time points to encourage participants to follow the training schedule and improve their adherence to the training program. The project also seeks to examine older adults' motivation to participate in cognitive training and investigate the effectiveness of optimally-timed tailored messaging to facilitate cognitive training.

This study uses data from a previous technology-based cognitive intervention study, which was conducted to investigate methods to improve adherence to a technology-based cognitive intervention (Harrell et al., 2021). The investigation enrolled 118 participants and was divided into structured and unstructured phases. In the structured phase of 12 weeks, participants were prescribed a training schedule involving gamified cognitive training tasks administered *via* a tablet. During the 6 week unstructured phase, participants were allowed to play as often as they liked. Results of the study showed that during the structured phase, message manipulations did not encourage adherence (Harrell et al., 2021). This motivates the need to study individual adherence patterns to determine older adults' upcoming compliance with real-time interventions and to evaluate how such an approach may improve their adherence to cognitive training programs.

The goal of the current study was to predict the participants' daily adherence to the training program based on their previous adherence patterns. We exploited deep learning models for this problem, as they automatically learn an informative set of features for a given dataset and have shown commendable performance in a variety of applications. A cursory examination of the data revealed that each participant exhibited a unique pattern of adherence. In order to capture the individual adherence characteristics, we decided to train a separate adherence prediction model for each participant, rather than the “one-size-fits-all” approach of training a single model for all participants. The optimal training window size was computed for each participant, and data augmentation was used to generate synthetic data and address the challenge of insufficient training data. The deep neural networks were trained to predict whether a given participant will meet the minimal adherence criterion on a given day (play for less than or greater than 10 min), based on previous playing pattern of the participant. This information will enable us to effectively identify participants who are most likely to be non-compliant to the training program. This, in turn, will help in designing an AI-based reminder system that can promote adherence by sending just-in-time tailored messages. To our knowledge, this research is the first of its kind to use advanced AI algorithms to predict older adults' daily adherence to cognitive training programs.

Academic research on the effectiveness of cognitive training programs aimed at combating cognitive decline has sparked a surge of interest in the past decade (Wolinsky et al., 2006; Jaeggi et al., 2008; Rebok et al., 2014; Simons et al., 2016; Zhang et al., 2019). While some studies have expressed doubt about the viability of such training programs (Talassi et al., 2007; Redick et al., 2013; Gray et al., 2021), others have shown that cognitive training is beneficial (Wolinsky et al., 2006; Talassi et al., 2007; Rebok et al., 2014). For example, the ACTIVE project's randomized study found that advanced cognitive training improved instrumental activities of daily living (IADL), resulting in the prevention and reduction of functional decline in older adults (Wolinsky et al., 2006; Rebok et al., 2014). Apart from the debate over whether cognitive training works, another crucial concern is whether older adults are willing to engage in such cognitive training. However, there is a scarcity of research that have examined how well people adhere to cognitive training and the elements that influence participants' adherence to the cognitive training programs (Sabaté, 2003; Turunen et al., 2019). Turunen et al. explored the effects of computer-based cognitive training (CCT) with older adults who had a higher risk of dementia. They discovered that prior computer use was the only factor linked with adherence in terms of the number of completed training sessions (Turunen et al., 2019). Harrell et al. explored the impact of positive and negative messages about brain health on adherence to home-based cognitive training interventions and discovered that positively-framed messages promoted greater adherence over negatively-framed messages (Harrell et al., 2021). They also investigated the factors that may correlate with participants' willingness to participate in cognitive training, such as age, belief in the efficacy of cognitive training, prior computer uses and technology proficiency. A better understanding of these factors can facilitate the prediction of adherence, allowing for more effective technology-based cognitive training. Machine learning algorithms have been previously used to predict adherence to medications (Koesmahargyo et al., 2020; Gu et al., 2021) and medical therapy procedures (Scioscia et al., 2021) with promising empirical results. Deep neural networks automatically learn informative feature representations and have depicted commendable performance in a variety of applications. In this research, we study the performance of deep learning techniques to predict adherence. These predictions can then be used to deliver tailored messages at optimal times to promote greater adherence to cognitive training. To the best of our knowledge, this is the first study to predict daily adherence to cognitive training programs in older adults using advanced machine learning techniques such as deep learning, data augmentation and adaptive window size estimation.

## 2. Materials

### 2.1. The mind frontiers program and gameplay

This project used the Mind Frontiers (Aptima Inc.) cognitive training software package, a Wild West-style Android video game application. The Mind Frontier cognitive training suite consists primarily of seven mini-video games modeled after measures of memory, attention, spatial processing, task-switching, reasoning ability, and problem-solving. Each of the games is supported by successfully implemented cognitive training programs in the psychology literature (Klingberg et al., 2002; Dahlin et al., 2008; Jaeggi et al., 2008; Karbach and Kray, 2009; Mackey et al., 2011; Harrell et al., 2021). Participants were instructed to play the games in a 12-week structured phase and in a 6-week unstructured phase following instructions detailed in the section below. After each game, participants received feedback and the difficulty of the game was adjusted based on their previous performance. The games were played on a Lenovo 10 tablet, and participants in the study were trained on both how to use the tablet and how to play each game.

### 2.2. Dataset

The dataset used in this study involves two phases (Harrell et al., 2021). Phase 1 was 12 weeks long and participants were asked to follow a prescribed schedule i.e., playing for 5 days out of 7, at 45 min a day. Phase 2 was unstructured and lasted 6 weeks, where participants were asked to play as frequently as they were willing to. In this study, we only analyzed the data collected during the structured phase, as adherence cannot be defined without the proposed game-playing instructions given in Phase 1. The study had 118 participants with an overall mean age of 72.6 years and a standard deviation of 5.5 years. Sixty-six percent of the participants were female with a mean age of 71.5, and thirty-two percent were male with the mean age of 75.0. There was no gender specific data for 2% of the participants.

Multiple cognitive assessments were recorded in the dataset, consisting of, but not limited to, technical competence, self-efficacy, subjective cognition, perceived benefits, and objective cognition (i.e., processing speed and memory). Training interaction details include information about interaction engagement, such as game duration, tasks performed, task levels, sessions, and the outcome of each task completed, as shown in Table 1. The cognitive training program contains 7 different tasks with 5 possible outcomes (i.e., defeat, stalemate, victory, abandonment, not yet completed).

**Table 1:** Data obtained through gamified cognitive training interactions.

**Category**	**Details**
Sessions	Sessions initiated by participants
Tasks	7 Tasks (WorkingMemory-Updating, Switching, Dual N Back, TowerOfLondon, PipeMania, FigureWeights VisualSpatial)
Task levels	Levels for tasks with maximum levels ranging from 16 to 58 for different tasks
Task outcomes	Outcome of each game. Total 5 possible outcomes (Defeat, Stalemate, Victory, Abort, Not Yet Finished)
Play/Interaction time	Duration of play time in number of seconds

To characterize participants' technology proficiency, participants were administered the Mobile Device Proficiency Questionnaire (MDPQ) (Roque and Boot, 2018). Proficiency varied widely with respect to the use of tablet computers (the technology platform for the current intervention). Scores for this measure range from a minimum score of 8 (indicating no experience or proficiency) to a max of 40 (indicating high proficiency using mobile devices such as tablets and smartphones). The average score for this sample was 27.10 (*SD* = 9.73). As expected, this average score was variable and substantially lower compared to college-aged samples (e.g., *M* = 38.4, *SD* = 1.7; from Roque and Boot 2018). To help reduce proficiency-related barriers to the adherence of the intervention, participants were given 1–2 h of training on how to use the tablet and the intervention software prior to the start of the intervention period. Participants were also given a custom user manual for how to operate the tablet and play each game within the intervention and were provided information on how to access to a technical support phone number.

We employed four time-dependent variables as predictors in our input data: (i) the length of time the participant played, (ii) the number of sessions, (iii) the highest level attained, and (iv) the number of tasks completed. Given the values of these predictors for a given participant for a specific number of days, our goal is to predict the play time of the participant for the following day.

### 2.3. Research question

**Figure 1:**
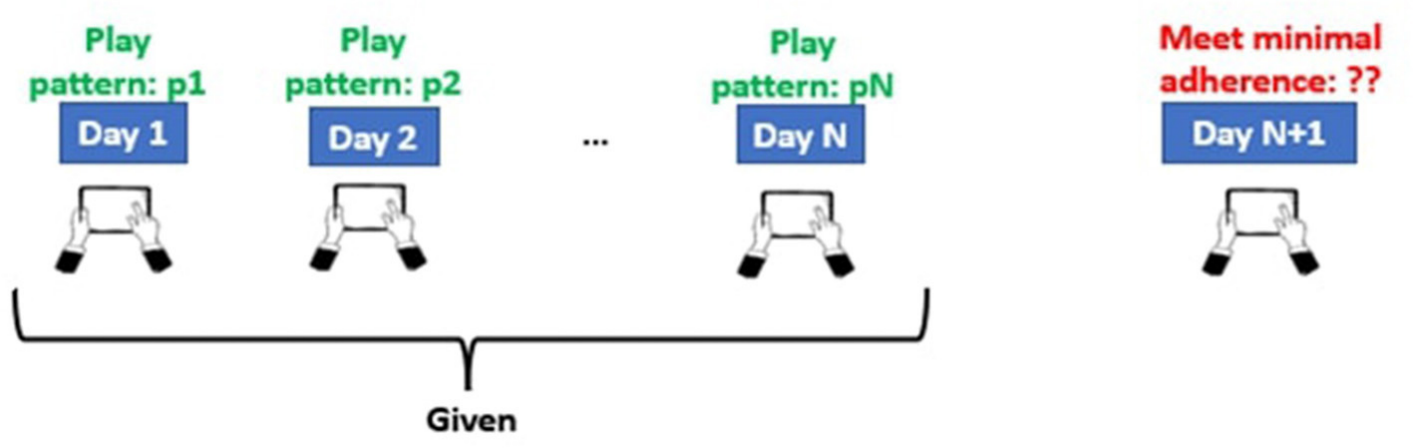
Research question: If the values *p*_1_, *p*_2_…, *p*_*n*_ of the time series at time stamp *t*_1_, *t*_2_, ..., *t*_*n*_ are given, can we predict the value at *t*_*n*+1_?.

The main purpose of this study was to use multivariate time series data acquired from neuropsychological game training data to determine whether participants will meet the minimum adherence criteria on day (N + 1), given the participants' N-day continuous play pattern, as depicted in Figure 1. For each participant, we split the data equally into training and testing, by taking the first 30 days of data for training and the next 30 days (31–60) of data for testing. This split was selected to strike a balance between the training and testing samples. Further, there is evidence in the psychology literature showing that the median time to develop different health-related habits asymptotes around 30 days (Lally et al., 2010). The proposed split can thus be useful to assess long-term adherence. The prediction of a particular day was made using the play information of prior N number of days where N is the window size. We used 4 time-dependent variables obtained through game interface interactions as our predictors, namely: (i) duration for which a participant played, (ii) number of sessions, (iii) max level reached and (iv) the number of tasks performed. The participants were said to be satisfying the *minimal adherence criterion if they played for at least 10 min on a given day*. This minimal adherence criterion was selected to ensure that the participants spent a reasonable amount of time playing the game, and to avoid cases where they may have accidentally opened the app and then backed out. Hence, this was considered as the threshold to define the two classes (adherent and non-adherent) in our study. Development of the predictive modeling system was particularly challenging because: (i) the amount of training data was limited (only 30 days of training data per participant), and (ii) we were attempting to predict the playtime for a particular day rather than the average over a period of time (1 or 2 weeks) where the margin of error can be comparatively lower due to averaging.

**Figure 2:**
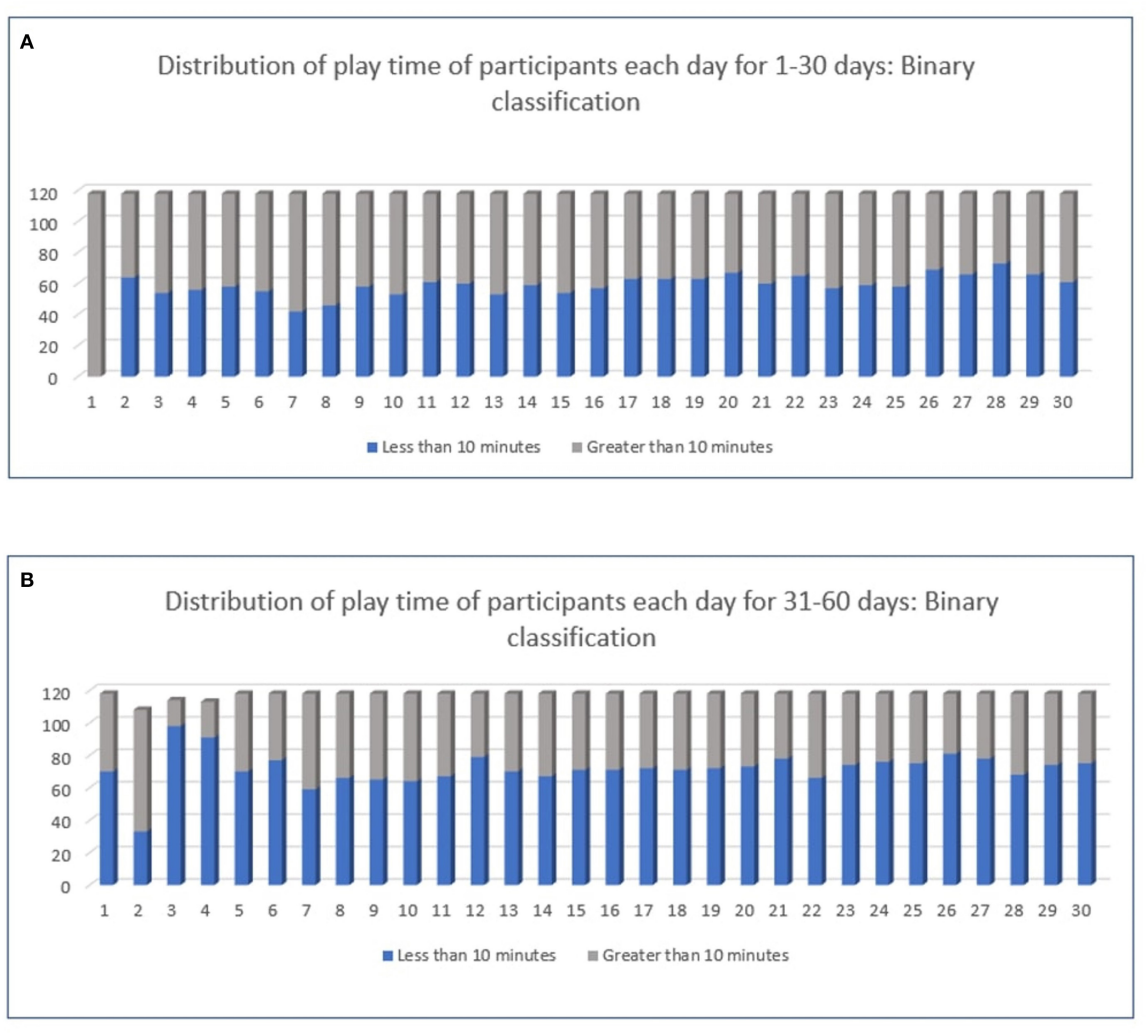
Distribution of play time of all the participants, **(A)** (top figure) in the first 30 days (training set) and **(B)** (bottom figure) the next 30 days (test set). The x-axis denotes the index of the day, and the y-axis denotes the number of participants. Best viewed in color.

Figures 2A,B show the distribution of play time of all the participants in the first 30 days (training set) and the next 30 days (test set). The length of the blue bar denotes the number of participants (out of 118) who played for less than 10 min on that day; the length of the gray bar denotes the number of participants who played for more than 10 min (i.e., those who met the minimal adherence criterion). We note that in days 31 to 60, the length of the blue bar is higher in many days, compared to that in the first 30 days. This means that participants tended to move from adherent to non-adherent in the later part of the study (that is, in the test phase). This demonstrates the necessity of our proposed approach in accurately identifying when a participant will fall off the training schedule, so that appropriate reminders can be issued accordingly.

## 3. Methods

Our proposed methodology consists of three main components (detailed below): (i) estimation of the optimal window size for each participant; (ii) data augmentation to address the challenge of limited training data per participant; and (iii) deep neural networks to learn patterns of adherence from the multivariate time series data. A separate deep model was trained for each participant, considering the individual differences in their play patterns; the final prediction accuracy was computed as the average accuracy over all the 118 participants.

### 3.1. Data processing and optimal window size estimation

Given a time series dataset, we can formulate a supervised learning problem using the sliding window method. In this method, relevant data from the recent past are passed as inputs to a predictive model, which is trained to predict the value of the response variable at the next time step. Since the play pattern of each participant is unique, we explored a method to determine the optimal window size for each participant and used it for that participant throughout the modeling process.

#### 3.1.1. Sliding window method

**Figure 3:**
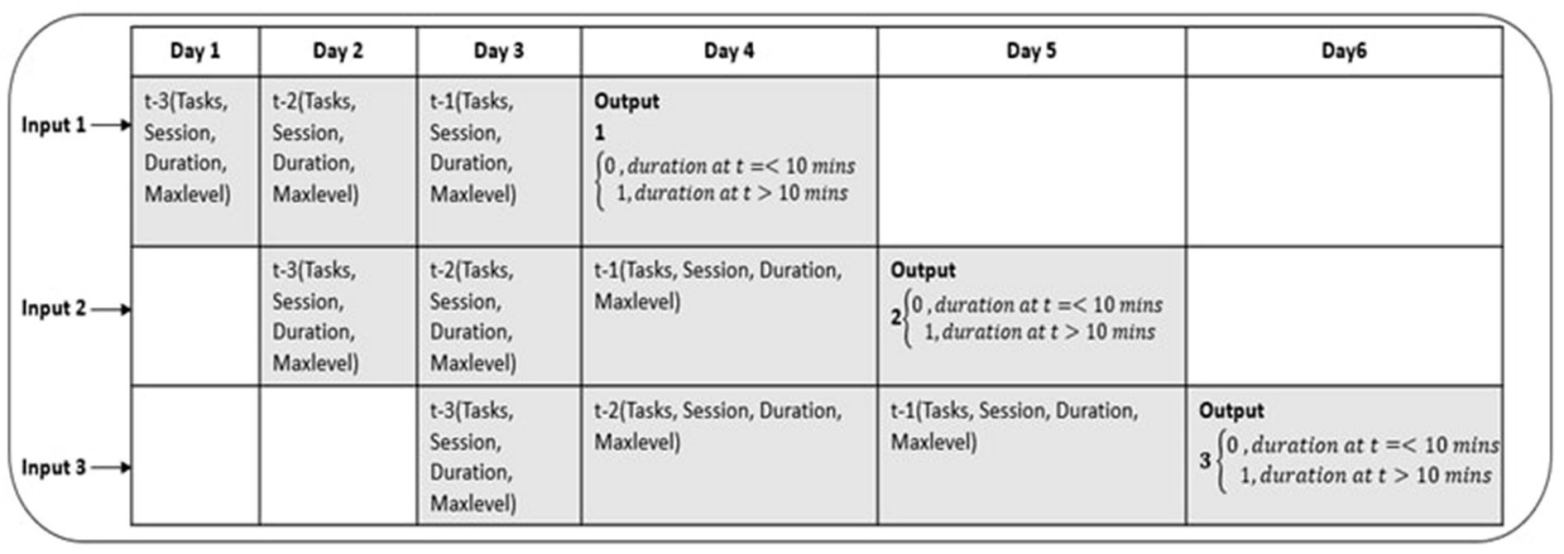
The figure illustrates structuring of time series data to fixed size windows (window size = 3 in this example).

The sliding/rolling window algorithm is a well-known technique for extracting subsequences from a long time series (Yu et al., 2014; Hota et al., 2017). The idea is to predict the value of the response variable at time t in a given time series, by feeding into the model values at time points (t-1), (t-2), (t-3) etc. as illustrated in Figure 3.

Before using a sliding window, we need to define two parameters: window size and sliding step. In this study, we exploited a technique to adaptively compute the optimal window size for each participant (based on their playing patterns), rather than use an arbitrary window size for all participants. This can potentially result in a better prediction model, as it captures the unique playing patterns of each participant. To this end, we used the seasonal decomposition of time series data to determine whether any cyclic variations or recurrence patterns can be obtained. The time difference, at which the most prominent cycle was observed, was used as the window size with a sliding step of 1. This is detailed below.

#### 3.1.2. Adaptive window size estimation

**Figure 4:**
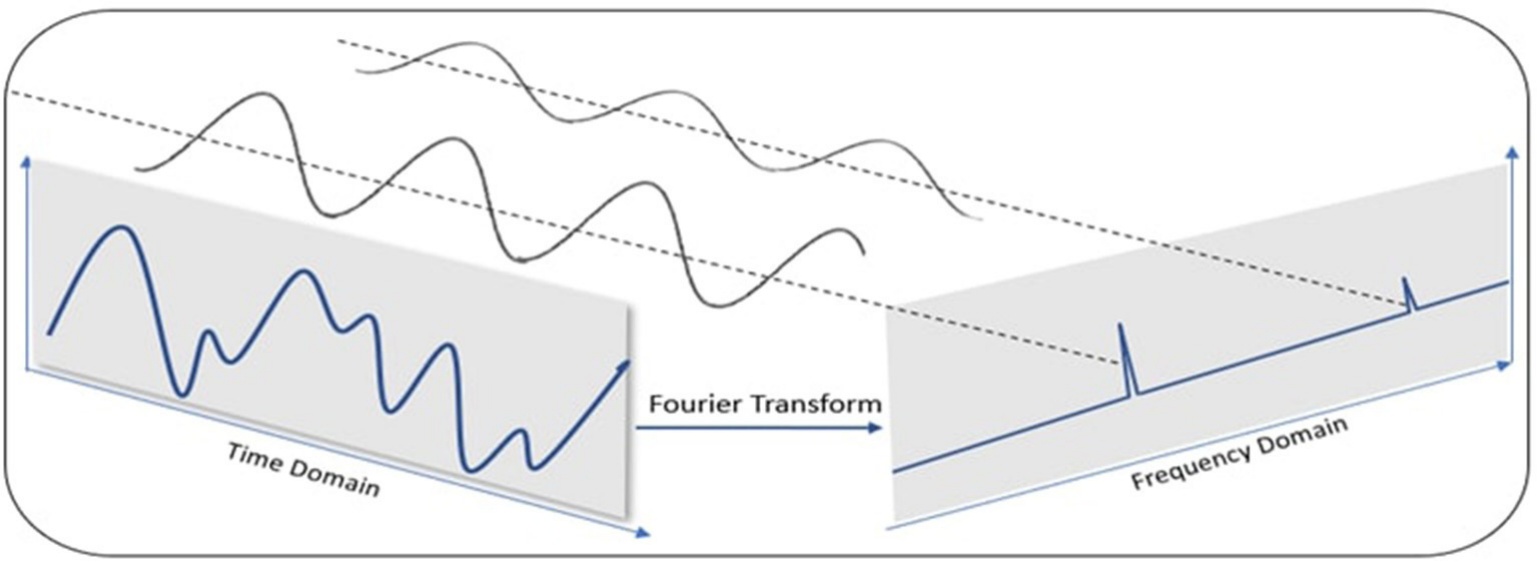
Illustration of Fast Fourier Transformation to convert data in time domain to frequency domain. In the time domain, the original signal is a superposition of signals with distinct frequencies. After applying FFT, we see corresponding strong frequencies in the spectrum.

Time series data can show different patterns, and it is often convenient to divide the time series into multiple components. Each component represents a basic category of patterns such as trend (general increase or decrease in average) and seasonality (a recurring cycle) (De Livera et al., 2011). We used the Fast Fourier Transform (FFT) to detect the presence of any seasonality in our time series data. The FFT allows us to transform a function of time and signal into a function of frequency and power (Musbah et al., 2019). This depicts the frequencies that make up the data in the original domain (time) and their relative strengths, as illustrated in Figure 4.

We first applied the FFT on play length attribute to transform the time series training data into the frequency domain and derive the corresponding amplitude and frequency. The highest amplitude frequencies represent seasonal patterns and the lowest amplitude frequencies represent noise. The inverse Fast Fourier Transform (IFFT) was then applied to the frequency with maximum amplitude to get the time interval for the most prominent cycle (periodic pattern). This cyclic period was used as the window size for the corresponding participant.

One of the challenges we faced in this research was the insufficient amount of training data per participant. We used the data we had and computed the FFT; our empirical results (shown in Tables 2–4) demonstrated the usefulness of optimal window size estimation through FFT computation. More training data per participant are likely to produce better results.

**Table 2:** Results using the CNN model.

CNN					
**Data augmentation technique**	**Precision**	**Recall**	**F score**	**AUC**	**Accuracy**
None	0.695	0.697	0.699	0.704	0.710
Jitter	0.696	0.713	0.731	0.724	0.713
Scaling	0.699	0.699	0.699	0.703	0.699
Time Warp	0.717	0.717	0.715	0.729	0.717
Jitter + Time Warp	**0.758**	**0.758**	**0.755**	**0.739**	**0.754**
Scaling + Time Warp	0.749	0.742	**0.755**	0.731	0.730

Values in bold indicate best performance.

**Table 3:** Results using the LSTM model.

LSTM					
**Data augmentation technique**	**Precision**	**Recall**	**F score**	**AUC**	**Accuracy**
None	0.706	0.714	0.704	0.708	0.706
Jitter	0.731	0.729	0.729	0.731	0.729
Scaling	0.706	0.703	0.703	0.728	0.703
Time warp	0.734	0.714	0.734	0.745	0.734
Jitter + Time Warp	0.748	**0.755**	**0.755**	0.739	**0.748**
Scaling + Time Warp	**0.749**	0.747	0.747	**0.748**	0.740

Values in bold indicate best performance.

**Table 4:** Results using the CNN-LSTM model.

CNN-LSTM					
**Data augmentation technique**	**Precision**	**Recall**	**F score**	**AUC**	**Accuracy**
None	0.722	0.719	0.714	0.713	0.718
Jitter	0.721	0.721	0.722	0.725	0.713
Scaling	0.713	0.713	0.713	0.720	0.717
Time warp	0.734	0.734	0.717	0.731	0.729
Jitter + Time Warp	0.735	0.726	**0.746**	**0.739**	0.724
Scaling + Time Warp	**0.750**	**0.752**	**0.746**	0.735	**0.735**

Values in bold indicate best performance.

### 3.2. Data augmentation for time series data

One of the fundamental challenges of this study was the scarcity of training data as only 30 days of training data were available for each participant. Deep neural networks are data-hungry and require a large amount of training data to attain good generalization performance. We exploited data augmentation techniques, which generate synthetic data samples to augment a given training set, to address the challenge of insufficient training data in this research. Data augmentation has been used in conjunction with time series data with promising empirical results (Um et al., 2017; Wen et al., 2020). Suppose our time series data is in the format *x* = *x*_1_, *x*_2_, ...*x*_*T*_ where the timestamp is *t* ∈ [1, 2…*T*]. We studied the performance of multiple data augmentation techniques, which are detailed below. We used a previous study to determine the values of the hyperparameters for data augmentation (Um et al., 2017).

#### 3.2.1. Jittering

A primitive but one of the most effective methods of transform-based data augmentation is injecting a small amount of noise/outliers in time series without changing the label, which makes it unique every time it is revealed to the model (Um et al., 2017; Iwana and Uchida, 2021). Jittering can be defined as:


(1) $$\begin{array}{l} x^{\prime }=x_{1}+\epsilon_{1},x_{2}+\epsilon_{2},\ldots x_{T}+\epsilon_{T} \end{array}$$

where ϵ is usually Gaussian noise added to the values at each time step t with ϵ ~ *N*(0, σ^2^). We used the hyperparameter σ as 0.01 in our studies. Since the produced patterns are distinct from the original by only a factor of noise, jittering has the potential to improve generalization and avoid overfitting.

#### 3.2.2. Scaling

Scaling resizes the time series by multiplying it by a scalar value which is usually generated from a Gaussian distribution (Um et al., 2017; Iwana and Uchida, 2021). It multiplies the entire time series by a parameter α, and can be represented as:


(2) $$\begin{array}{l} x^{\prime }=\alpha_{1}x_{1},\alpha_{2}x_{2},...\alpha_{T}x_{T} \end{array}$$

where the scaling parameter α is determined from a Gaussian distribution α ~ *N*(1, σ^2^). We used σ as 0.1 in our studies.

#### 3.2.3. Time warping

Data augmentation using time warping techniques deform a pattern in the temporal dimension using a smooth warping path (Um et al., 2017; Iwana and Uchida, 2021). The augmented time series is represented as:


(3) $$\begin{array}{l} x^{\prime }=x_{\tau \left(1\right)},x_{\tau \left(2\right)},...x_{\tau \left(T\right)} \end{array}$$

where τ(.) is a warping function that perturbs the time steps based on a smooth curve. This curve is described by a cubic spline which is formed by using a cubic polynomial in an interval between two successive knots (Li et al., 2014). The knot height is derived from a Gaussian distribution *N*(1, σ^2^). We used σ as 0.2 in our experiments.

#### 3.2.4. Stacking scaling/jittering with time warping

The aforementioned data augmentation techniques can be combined to devise new augmentation techniques (Um et al., 2017). Depending on the application in question, the combination of two or more of these techniques can potentially improve the generalization performance. In our experiments, applying time warping in addition to scaling/jittering significantly improved the results.

We increased the training data by 5 folds using each of the above mentioned data augmentation techniques and compared their performance in the empirical studies detailed below.

### 3.3. Deep neural networks for time series data classification

Deep neural networks have been successfully employed to address the problem of time series data classification. It has proven to be an efficient solution because it can automatically assess the temporal dependencies that occur in time series data and learn a discriminating set of features accordingly. We used three deep model architectures that have depicted commendable performance in time series classification namely, Convolution Neural Network (CNN), Long Short-Term Memory Network (LSTM), and a combination of CNN and LSTM.

#### 3.3.1. Convolution neural network

Convolutional Neural Networks have depicted impressive performance in a variety of time series data classification applications (Chen et al., 2016; Kim, 2017; Zhao et al., 2017; Liu et al., 2018; Ismail Fawaz et al., 2019). The model uses the convolution operation to extract meaningful features from the raw data. In 1D convolution, the kernel moves in one direction from the beginning of a time series toward its end. If we have input vector f of length n and a kernel *g* of length *m*, the convolution *f* * *g* of *f* and *g* is defined as follows:


(4) $$\begin{array}{l} \left(f*g\right)\left(i\right)=\overset{m}{\underset{\left(j=1\right)}{\sum }}g\left(j\right).f\left(i-j+\frac{m}{2}\right) \end{array}$$

**Figure 5:**
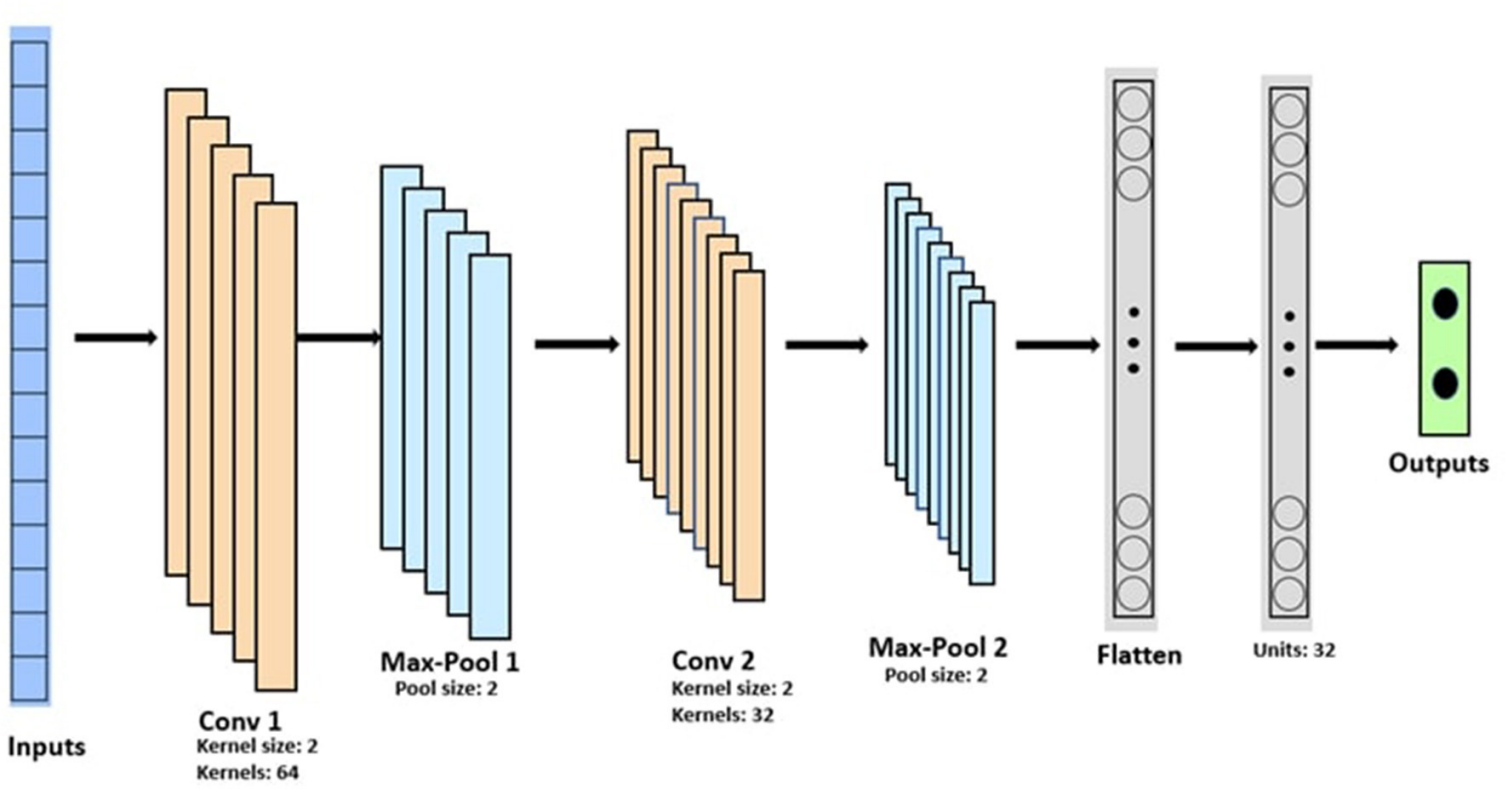
Convolution Neural Network architecture. Conv 1 is the first 1-D Convolution layer with 64 filters of size 2, followed by a Max-Pool layer (Max-Pool1) with a pool size of 2, stacked with a second block of 1-D Convolutional layer (Conv 2) and Max-Pool layer (Max-Pool2), followed by a flatten layer, a fully connected layers with 32 neuron units and the output layer with sigmoid activation function.

We used two convolution blocks consisting of a convolution layer and a maximum pooling layer. Maximum pooling layer moves a pool of predetermined size across the input and calculates the maximum of the region. These blocks are followed by a dense and an output layer. Figure 5 shows the architecture of the CNN used in this research.

#### 3.3.2. Long short-term memory network

Previous studies have shown the effectiveness of recurrent neural networks (RNNs), in particular, long short-term memory networks (LSTMs) for time-series prediction, due to their capability for learning from long observation sequences (Siami-Namini and Namin, 2018; Pham, 2021). Instead of neurons, LSTM networks have layered blocks of memory. Each block contains a set of gates that manage the state and output of the block. The first is a forget gate *f*_*t*_ that lets the model choose whether to remember the previous timestamp information or if it is irrelevant and can be forgotten. Second is the input gate *i*_*t*_ that allows the cell to gather new data from the input. Finally, the output gate *o*_*t*_, provides the updated information from the current timestamp to the following one. The equations for the forward propagation of a LSTM cell are shown in Equations (5) and (6) (Hochreiter and Schmidhuber, 1997):


$$\begin{array}{l} f_{t}=\sigma \left(w_{f}\left[h_{t-1},x_{t}\right]+b_{f}\right) \end{array}$$

$$\begin{array}{l} i_{t}=\sigma \left(w_{i}\left[h_{\left(t-1\right)},x_{t}\right]+b_{i}\right) \end{array}$$

(5) $$\begin{array}{l} o_{t}=\sigma \left(w_{o}\left[h_{\left(t-1\right)},x_{t}\right]+b_{o}\right) \end{array}$$

where σ is the sigmoid function *w*_*f*_, *w*_*i*_, *w*_*o*_, *b*_*f*_, *b*_*i*_, *b*_*o*_ are the weights and biases for the respective gates, *h*_*t*−1_ is output of the previous LSTM block at timestamp *t* − 1 and *x*_*t*_ is input at current timestamp. Further, the cell state *c*_*t*_, candidate cell state $\tilde{c_{t}}$ and output *h*_*t*_ of LSTM are calculated as shown in Equation (6). At any timestamp, the cell state evaluates what to forget from the previous state (*i*.*e*., *f*_t_*c_*t*−1_) and what it needs to consider from the current timestamp $\left(i.e.,i_{t}*\tilde{c_{t}}\right)$


$$\begin{array}{l} \tilde{c_{t}}=tanh(w_{c}[h_{t-1},x_{t}]+b_{c} \end{array}$$

$$\begin{array}{l} c_{t}=f_{t}*c_{t-1}+i_{t}*\tilde{c_{t}} \end{array}$$

(6) $$\begin{array}{l} h_{t}=o_{t}*tanh\left(c_{t}\right) \end{array}$$

**Figure 6:**
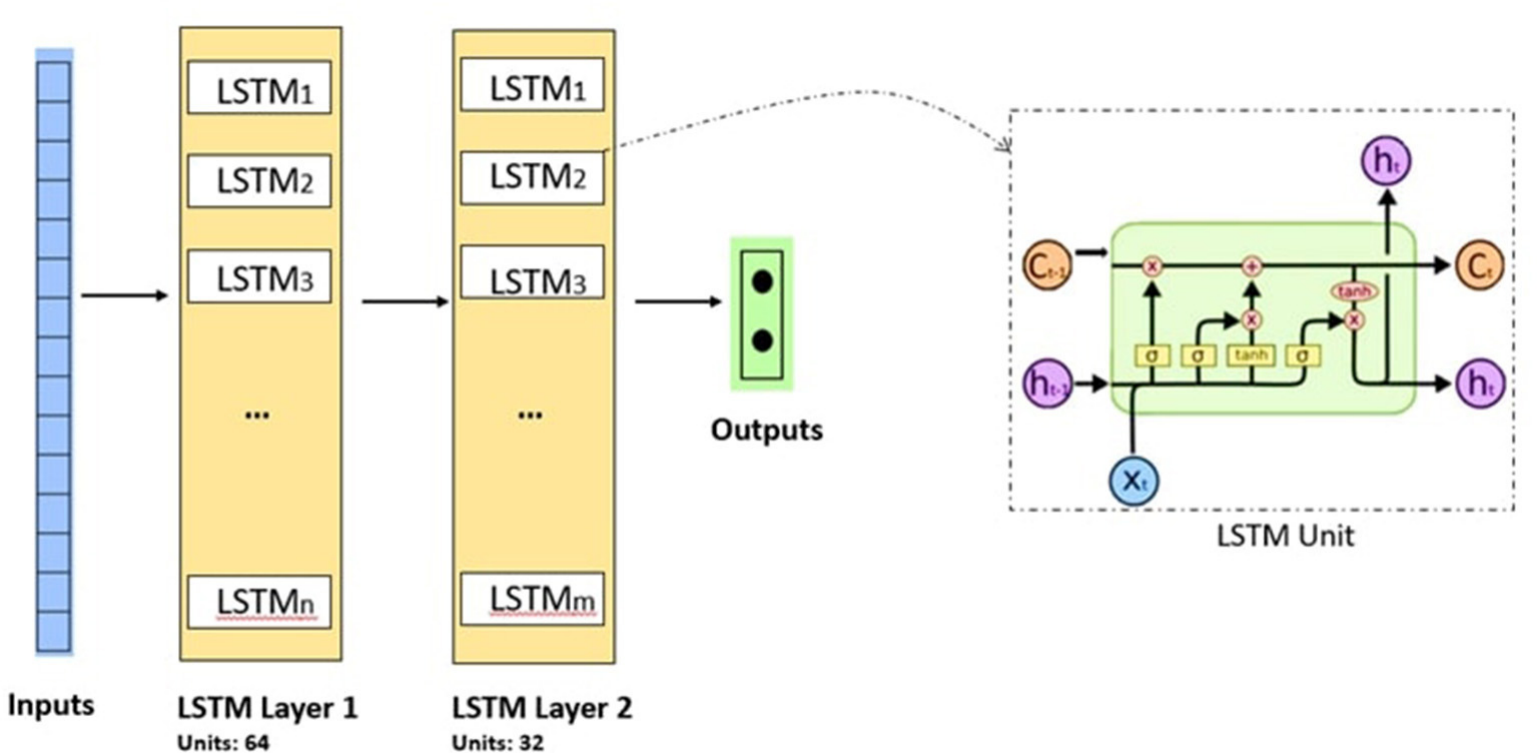
Convolution Neural Network architecture. Conv 1 is the first 1-D Convolution layer with 64 filters of size 2, followed by a Max-Pool layer (Max-Pool1) with a pool size of 2, stacked with a second block of 1-D Convolutional layer (Conv 2) and Max-Pool layer (Max-Pool2), followed by a flatten layer, a fully connected layers with 32 neuron units and the output layer with sigmoid activation function.

Figure 6 shows the general architecture of the LSTM model used in this study.

#### 3.3.3. CNN-LSTM hybrid network

**Figure 7:**
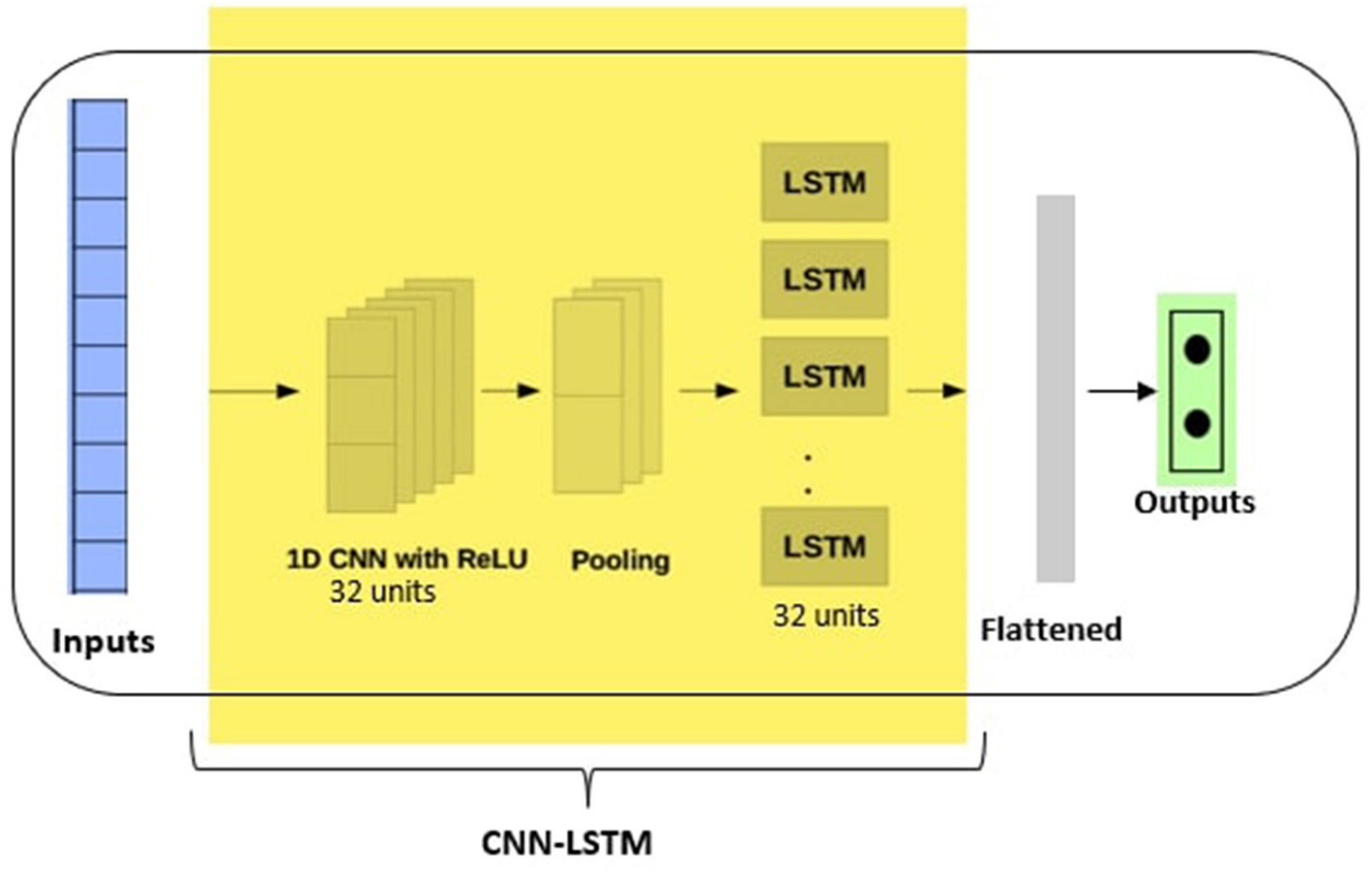
CNN-LSTM hybrid architecture with 32 CNN filters and 32 LSTM units and the output layer with sigmoid activation function.

CNN models can be used with LSTM backends. The CNN is first used to interpret the subsequences of the inputs, that are then provided together as a sequence to the LSTM model for interpretation. This hybrid model is called CNN-LSTM. The CNN-LSTM model has been studied to analyze 1-D time series data, with promising empirical results (Liu et al., 2017; Lu et al., 2020; Mutegeki and Han, 2020). We therefore included this model in our empirical study. To use such a deep model architecture, the first step is to divide the input sequence into subsequences that can be processed by the CNN model. This subsequence interpretation is then provided as an input to the LSTM model for processing. Figure 7 shows the architecture of the CNN-LSTM model used in our study.

### 3.4. Experimental setup

**Figure 8:**
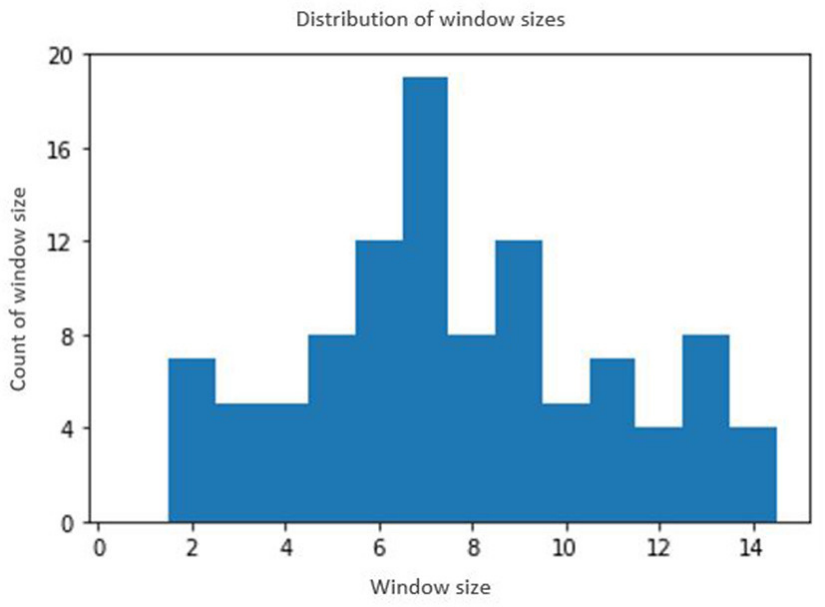
Distribution of window sizes for 118 participants. The x-axis denotes the window size and y-axis denotes the number of participants with the corresponding window size.

**Figure 9:**
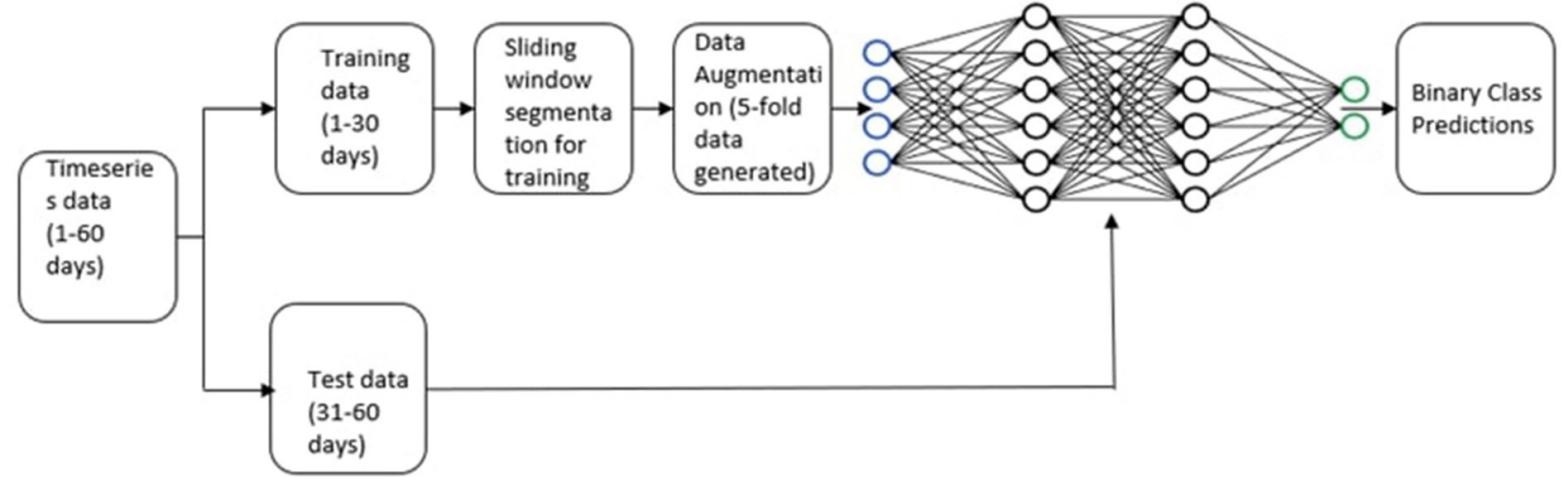
Experimental framework for multi-variate time series classification.

For each of the 118 participants, the data for the first 30 days constituted the training data and the data for the next 30 days constituted the test data. The optimal window size was determined for each participant using the method described in Section 3.1. Depending on the size of the window, the training sample size varies for each participant. For instance, if the window size for a participant is 3, then 3 days of data (day 1 through day 3) constitutes one training sample and the label is given by the play time on day 4. By moving the window by one, the subsequent training sample constitutes data from day 2 through day 4 whose label is given by the play time on day 5, and so forth. This will give us a total of 30 samples for training for this participant (since we are using the first 30 days of data for training). The test data is prepared in a similar manner. For the given example, the first testing sample will begin on day 31 and continue through day 33 and our goal will be to predict the play time on day 34, and so forth. Figure 8 shows the distribution of window size across all the 118 participants. Data augmentation techniques were then applied to increase the training data by 5 folds from the original time series, making the total data size 6 times the original number. The deep learning models were trained using the original, as well as augmented data. The label was acquired by the playtime of the participant on the following day, and a 10-min threshold was used on the time of play to binarize the classes (adherent and non-adherent). The goal was to train deep models to predict the adherence class on a particular day for a given participant, by using his/her playing data from the recent past, as determined by the sliding window method. A separate, personalized deep neural network was trained for each participant to capture the individual playing patterns, rather than attempting to fit a single model for all participants. The final accuracy was computed as the average accuracy over all the participants. Figure 9 shows the entire modeling process.

### 3.5. Evaluation metrics

Since we are attempting to solve a binary classification problem, we used five evaluation metrics in this research: precision, recall, F-score, area under curve and accuracy. These are detailed below:

- **Precision:** Precision is the ratio of the number of true positive predictions to the total number of positive predictions (Goutte and Gaussier, 2005). High precision implies low false positive rate.

(7) $$\begin{array}{l} Precision=\frac{TruePositives}{TruePositives+FalsePositives} \end{array}$$

- **Recall (Sensitivity)**: Recall is the ratio of the number of true positive predictions to the number of samples that actually belong to the positive class (Goutte and Gaussier, 2005). High recall relates to low false negatives.

(8) $$\begin{array}{l} Recall=\frac{TruePositives}{TruePositives+FalseNegatives} \end{array}$$

- **F1-score:** F1 Score is the harmonic mean of precision and recall. For this measure to achieve a high value, both precision and recall need to be high (Sokolova et al., 2006). It is computed as:

(9) $$\begin{array}{l} F1Score=\frac{2*\left(Recall*Precision\right)}{Recall+Precision} \end{array}$$

- **Area Under Curve (AUC):** A receiver operating characteristic (ROC) curve is a graph that shows the performance of a threshold-based classifier at different classification thresholds (Bradley, 1997). This curve plots the false positive rate (on the x-axis) against the true positive rate (on the y-axis). AUC computes the entire two-dimensional area under the ROC curve. A higher value of the AUC implies a better model at differentiating between the positive and negative classes.
- **Accuracy:** Accuracy is the ratio of the number of correct predictions to the total number of predictions. It is computed as:

(10) $$\begin{array}{l} Accuracy=\\ \frac{TruePositives+TrueNegatives}{TruePositives+TrueNegatives+FalsePositives+FalseNegatives} \end{array}$$

## 4. Results

Tables 2–4 show the performance of convolutional neural networks, long-term memory recurrent neural networks, and hybrid CNN-LSTM networks, respectively. We explored five data augmentation techniques (jitter, scaling, time warping, jitter + time warping, scaling + time warping) and compared their performances using the five evaluation metrics.

We note that the data augmentation techniques improved the generalization performance consistently for all the three deep model architectures studied. The best performance achieved for all the experiments was using some form of data augmentation. The combination of jitter + time warping and scaling + time warping depicts the most promising performance among all the data augmentation techniques and achieved approximately 75% accuracy, AUC and F-score. Particularly, for the CNN model (Table 2), the highest F-score of **75.5%** and the highest accuracy of **75.4%** were both obtained using jitter with time warping as the data augmentation technique. Scaling with time warping also achieved the same F-score. The LSTM model (Table 3) also furnished the highest F-score of **75.5%** and the highest accuracy of **74.8%** using jitter and time warping. For the CNN-LSTM hybrid model, the highest F-score **(74.6%)** and the highest accuracy **(73.5%)** were both obtained using scaling with time warping. Jitter with time warping also produced the same F-score. These results corroborate the potential of deep neural networks and advanced machine learning techniques like data augmentation and adaptive window size estimation to learn informative feature representations from time series data, for the challenging task of predicting daily adherence to cognitive training programs in older adults.

## 5. Discussion and conclusions

The goal of our ongoing project *Adherence Promotion with Person-Centered Technology (APPT)* is to develop an adaptive reminder system to increase adherence to mobile-based cognitive training in older adults, with the ultimate goal of promoting early detection and prevention of age-related cognitive decline. Instead of sending generic reminder messages, our objective is to send tailored messages to each participant based on their individual preferences and routine, to better motivate them to adhere to the training schedule. Further, we are interested in identifying the optimal time points when the reminder messages will have maximal impact for each participant based on their daily schedules, rather than sending them at arbitrary time points during the day. Designing such a just-in-time reminder system necessitates an accurate understanding of the training patterns of each participant, and a mechanism to accurately predict when they are most likely to fall off their training schedules. The research presented in this paper summarizes our findings of using state-of-the-art artificial intelligence (AI) algorithms for predicting adherence to a cognitive training program. We used game data from the Mind Frontier Program to predict whether a particular participant would play for a particular period on a given day, based on their past playing behavior. To the best of our knowledge, this is the first research effort to use advanced machine learning and deep learning techniques to predict daily adherence to cognitive training programs in older adults. Our significant findings in this research can be summarized as follows: (i) deep neural networks are effective in learning informative feature representations from time series data to predict daily adherence. Our empirical studies demonstrated more than 75% F-score using deep learning models. Koesmahargyo et al. (2020) and Scioscia et al. (2021) studied the performance of boosting and SVM models for adherence prediction. Gu et al. (2021) also studied the performance of several machine learning models and a deep learning MLP model for predicting adherence. In contrast, we studied the performance of advanced deep learning models (CNN, LSTM and CNN-LSTM) for predicting daily adherence to cognitive training. Note that predicting the adherence for a particular day is much more challenging than predicting the average adherence over a period of time (1 week or 2 weeks), where the margin of error can be much lower due to averaging. (ii) Each participant has a unique playing pattern; further, only 30 days of data were available for training for each participant. Thus, computing the window size adaptively for each participant and using data augmentation techniques to generate synthetic training data, are crucial in training reliable deep neural networks for daily adherence prediction. We conducted an experiment, where we used a constant window size of 3 for all the participants and no data augmentation. Using the same deep learning models, the highest accuracy achieved was approximately 70%, showing the usefulness of optimal window size computation and data augmentation techniques. To the best of our knowledge, this is the first research effort to use such advanced machine learning and signal processing techniques to predict daily adherence to cognitive training programs in older adults.

The deep neural networks learn informative feature representations; data augmentation techniques augment the training data in order to train robust deep models with good generalization capabilities; optimal window size estimation computes the appropriate window size for each participant, which helps us in determining how much data we should observe to make a prediction for that participant. Thus, the principles of feature learning, optimal window size estimation and data augmentation are strongly inter-related and work in tandem toward the common goal of accurately predicting daily adherence to medication reminders for older adults. The proposed methodologies can be used to determine when a participant's adherence is going to drop during the course of the training program. We hope this research will be a step toward the development of a novel AI-based reminder system to encourage participants to adhere to their schedules in any mobile-based cognitive training program and can provide a useful approach for enhancing adherence to other health-related behaviors (e.g., exercise, medication consumption).

As mentioned earlier, one of the challenges we faced in this research was the limited amount of training data per participant. While data augmentation techniques have demonstrated tremendous promise in addressing this challenge, a larger amount of training data can potentially improve the generalization performance of the deep models, particularly the more complicated architectures like hybrid CNN-LSTM, which has a much larger number of parameters to be trained compared to a CNN or LSTM.

We used 4 predictors for each participant (duration of play, number of sessions, max level reached and number of tasks) in our experiments. The dataset also contains demographic information of the participants; however, since the training and test data for each model come from the same participant, the demographic information remains constant and hence, could not be used as predictors.

### 5.1. Future work

**Figure 10:**
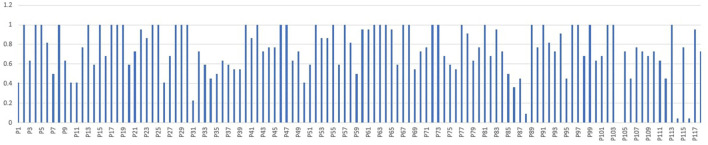
Plot of adherence prediction accuracy for each of the 118 participants in our study.

As part of future work, we plan to explore strategies to further improve the prediction accuracy of the deep learning models. Figure 10 shows a detailed plot of the adherence prediction accuracy for each of the 118 participants in our study. We note that some participants' gameplay behaviors were easy to predict (almost perfect or near-perfect prediction accuracy) while others were much more difficult. We plan to explore two strategies to boost the performance of the deep models corresponding to the latter group of participants: (i) generate more synthetic data using data augmentation to increase the number of training samples; (ii) use advanced variants of LSTMs, such as GRUs and attention-based LSTMs, which have depicted impressive empirical performance. The attention-based LSTMs focus on specific or interconnected sub-problems (Li et al., 2019), which is achieved by shifting the weights within the network. Furthermore, there has been an increase in the use of hybrid models such as Adaboost-LSTM (Sun et al., 2018) that often perform superior to the corresponding single predictive model for predicting time series data. We plan to explore these in our research.

Further, domain adaptation or transfer learning techniques are instrumental in developing a model for a target domain of interest, where training data is scarce, by leveraging ample labeled training data in a source domain, under the constraint of a probability distribution difference between the two domains (Pan and Yang, 2009; Ganin and Lempitsky, 2015). Multi-source domain adaptation, which contains multiple source domains, and a single target domain has also attracted significant research attention (Chattopadhyay et al., 2012; Sun et al., 2015). As part of future research, we plan to study these techniques to address the challenge of insufficient training data. In our application, a given participant of interest constitutes the target domain, while each of the other participants constitute a source domain. There is an inherent disparity among the different domains, as each participant has his/her own unique pattern of training. Domain adaptation algorithms can address this domain disparity, so that training data from other participants can be used to develop a prediction model for the given target participant. This can potentially improve the prediction accuracy due to the additional training data per participant.

## Data availability statement

The raw data supporting the conclusions of this article will be made available by the authors, without undue reservation.

## Ethics statement

The studies involving human participants were reviewed and approved by Florida State University Human Subjects Committee. The patients/participants provided their written informed consent to participate in this study.

## Author contributions

AS: conceptualization, methodology, software, formal analysis, validation, and writing—original draft. SC: conceptualization, methodology, supervision, and writing—review and editing. ZH and ST: conceptualization and writing—review and editing. SZ and ML: writing—review and editing. NC: conceptualization, methodology, project administration, and writing—review and editing. NR and EH: data curation. WB: conceptualization, methodology, funding acquisition, project administration, and writing—review and editing. All authors contributed to the article and approved the submitted version.

## Funding

This work was supported by the National Institute on Aging grant R01AG064529. This study was also partially supported by University of Florida-Florida State University Clinical and Translational Science Award funded by National Center for Advancing Translational Sciences under Award Number ULITR001427.

## Conflict of interest

The authors declare that the research was conducted in the absence of any commercial or financial relationships that could be construed as a potential conflict of interest.

## Publisher's note

All claims expressed in this article are solely those of the authors and do not necessarily represent those of their affiliated organizations, or those of the publisher, the editors and the reviewers. Any product that may be evaluated in this article, or claim that may be made by its manufacturer, is not guaranteed or endorsed by the publisher.

## Author disclaimer

The content is solely the responsibility of the authors and does not necessarily represent the official views of the NIH.
